# Assessing the proxy response bias of EQ–5D-3 L in general population: a study based on a large-scale representative household health survey using propensity score matching

**DOI:** 10.1186/s12955-020-01325-z

**Published:** 2020-03-18

**Authors:** Ying Liang, Tianle Che, Haiyue Zhang, Lei Shang, Yuhai Zhang, Yongyong Xu, Lingxia Guo, Zhijun Tan

**Affiliations:** 1 Department of Health Statistics, Fourth Military Medical University, Xi’an, Shaanxi Province China; 2 Ministry of Education Key Lab of Hazard Assessment and Control in Special Operational Environment, Fourth Military Medical University, Xi’an, Shaanxi Province China; 3 Center of Health Statistics, Health General Office of Shaanxi Province, Xi’an, Shaanxi Province China

**Keywords:** Proxy response bias, Health-related quality of life, EQ-5D-3 L, Propensity score matching

## Abstract

**Background:**

Proxy respondent-someone who assists the intended respondent or responds on their behalf-are widely applied in the measurement of health-related quality of life (HRQL). However, proxies may not provide the same responses as the intended respondents, which may bias the findings.

**Objectives:**

To determine whether the use of proxies is related to socio-demographic characteristics of the intended respondent, and to assess the possible proxy response bias of Chinese version of EQ-5D-3 L in general population.

**Methods:**

A cross-sectional study based on a provincially representative sample from 2013 National Health Service Survey (NHSS) in Shaanxi, China was performed. HRQL was measured by Chinese version of EQ-5D-3 L. Propensity score matching (PSM) was used to get matched pairs of self-reports and proxy-reports. Before and after PSM, univariate logistic and linear models including the indicator of proxy response as the only independent variable, were employed to assess the possible proxy response bias of the dimensional and overall health status of EQ-5D-3 L respectively.

**Results:**

19.9% of the responses involved a proxy. Before PSM, the proxy-report group was younger in age and reported less unhealthy lifestyle, lower prevalence of disease, and less hospitalization than the self-report group. After PSM, it showed that the proxy-report group was statistically more likely to report health problem on each dimension of EQ-5D-3 L, with odds ratios larger than one comparing with self-report group. The means of EQ-5D-3 L index and EQ VAS of proxy-report group were 0.022 and 0.834 lower than self-report group.

**Conclusions:**

Significantly negative proxy response bias was found in Chinese EQ-5D-3 L in general population, and the magnitude of the bias was larger in physical dimensions than psychological dimensions after using PSM to control confounders.

## Introduction

Health-Related Quality of Life (HRQL) is known as an important component of health evaluation in addition to conventional objective indicators, such as morbidity, mortality and clinical measurements [1–4]. In order to ensure adequate sample size and reduce the selection bias for the survey, proxies (e.g., family, professional caregiver, friend, relative) are usually allowed to substitute for the intended respondents who are unavailable (e.g., institutionalized or hospitalized) or unable (e.g., physical or cognitive impairments) to complete the questionnaire on their own behalf [5]. However, this may bring significant proxy response bias into HRQL measurement [5–9].

EQ-5D is a generic instrument widely applied for measuring HRQL and health technology assessment (HTA) in many countries [10–14]. In China Guidelines for Pharmacoeconomic Evaluations, one of the most important HTA guidelines in China, EQ-5D is one of the four recommended preferred outcome for QoL [15]. To date, extensive studies have assessed the inter-rater reliability between self-report and proxy-report EQ-5D [16–28]. The inter-rater reliability is primarily measured using *precision*—the strength of agreement between proxy and patient responses, and *bias*—systematic difference in proxy response [29]. These existing studies primarily investigated the inter-rater reliability on specific population, such as children [19, 27], older adults [24, 27, 28], dementia [16, 18, 21, 23–25], stroke [17], prolonged mechanical ventilation [20], intensive care [22], and vascular cognitive impairment [25]. In general, most of these studies showed that proxies were inclined to under-estimate the patients’ health conditions, and proxy-reports and patient-reports did not agree and were inconsistent in terms of proxy type (e.g. spouse, relatives, or health professionals, etc.), observability of the domains or characteristics of the patients.

There are a number of limitations to the existing studies into proxy response bias in EQ-5D. The most important one is that most studies were done with paired proxy-patient populations, in which each intended respondent is paired with a proxy and both the proxy and the intended respondent report EQ-5D indicating that the proxies are not necessary, even though the proxy response bias might be “real”. In practice, however, proxy responses are only necessary when the responses of the intended respondents are not available. To date, no studies in EQ-5D-3 L evaluated proxy response bias by using unpaired study design. Another important limitation is that most studies cover relatively small samples of population with specific health problems. But proxy response bias takes on great importance in large representative surveys, such as the Behavioral Risk Factor Surveillance System (BRFSS) [30, 31], and the National Health Interview Survey (NHIS) [32], from which the conclusion can be generalized to the general population, and are more valuable for decision-making in public health. In addition, small sample size is not sufficient to examine the incidence of proxy responses among different demographic groups, thus making it difficult to understand how often “real” proxy responses are needed and used.

Investigating the “real” proxy response bias of HRQL in a large representative survey can be complicated, because the actual health profiles of the intended respondents who uses proxy are not available. Studies have shown that HRQL is affected by numerous factors, such as age, marital status, health behavior, and chronic disease morbidity [33–38]. In order to accurately measure the proxy response bias of HRQL in a cross-sectional survey, many confounding factors must be controlled simultaneously. Propensity score matching (PSM) is a technique that usually applied to mimic randomized controlled trials (RCTs), which can minimize the bias caused by confounding factors, and similar results to RCTs can be obtained [39, 40], therefore PSM is applicable to address the limitations described above [5, 7, 29].To date, there is no study investigating proxy response bias of the Chinese version of EQ-5D-3 L in general population. Based on a large representative survey of general population, this study has two objectives: (1) to determine whether the use of proxies is related to socio-demographic characteristics of the intended respondents; (2) to evaluate the presence, direction and magnitude of possible proxy response bias in the EQ-5D-3 L.

## Methods

### Data source

#### National Health Service Survey

The National Health Services Survey (NHSS) is one of the most influential health survey in China [41, 42]. The data was obtained from the 2013 National Health Services Survey (NHSS) in Shaanxi province, which has been conducted every 5 years since 2003. Now, it is one of the largest and influential health surveys in this area. The four-stage cluster unequal probability sampling method was used to select a provincially representative sample. Subject to the sampling design reported in our previous study [43], a total of 32 counties (districts), 160 townships (streets), 320 rural or residential committees, 20,700 households and 57,529 people were selected. The survey questionnaire included more than 200 questions relating to the area of socio-economic characteristics, health status, health risks and health service needs and utilization. Household interviews were used to collect the data. It allows family members familiar with the recent situation of the intended respondents to take the interview, as proxies. Rigorous quality control measures were taken at every stage to ensure the quality of the survey, and the good quality was also evidenced by its Myer’s blended index, which is a method to evaluate the quality of the survey. The Myer’s blended index shows a range of 0–99. Zero denotes a consistency in age distribution of the sample and of the population, and 99 denotes that the age of the samples ends with the same number. The Myer’s blended index greater than 60 indicates that the investigated sample has a serious age preference. The Myer’s blended index of the sample in this study was only 1.3. Participants aged 15 and above were included and all individuals with missing values among EQ-5D-3 L and other analysis variables were excluded. Finally, a total of 44,134 individuals were chose in the analysis.

#### EQ-5D-3 L instrument

EQ-5D-3 L has gained widespread popularity for it is easy to be administered, scored, and interpreted, especially in large-scale face-to-face health interview surveys. EQ-5D-3 L has been included in NHSS since 2008. EQ-5D-3 L consists of two components, the EQ-5D-3 L health descriptive system and EQ VAS. The former, EQ-5D-3 L health descriptive system, is comprised of 5 dimensions, including mobility (MO), self-care (SC), usual activities (AC), pain/discomfort (PD), and anxiety/depression (AD). Each dimension consists of three categories, namely, no problems, some problems, and extreme problems. In general population, the proportion of reporting extreme problems (the third level) in each dimension of EQ-5D-3 L is very low [42]. To simplify the expression of the proportion of reporting health problems and to improve the robustness of the estimations of the proxy response bias, the original response outcome in each dimension was transformed into two categories (reporting no problem and reporting any problem). Chinese time trade-off values were used to calculate the EQ-5D-3 L index based on these five dimensions [44]. The EQ VAS is a 20-cm long vertical visual scale, with the highest score of 100 corresponding to “the best health you can imagine”, while the bottom score of 0 corresponding to “the worst health status you can imagine”.

#### Other important variables

The following question was used to identify proxy responses:

*Q31. Who answers the following questions (judged by the investigator)?

i. Answer by yourself
ii. Reply by others

Thirteen covariates were used to calculate propensity score, including socio-demographic factors, health behavior, and health status associated factors. Continuous covariates and categorical covariates with too many levels were reclassified, such as age (15–44, 45–64, and 65+ years, which representing the young adult, the middle-aged, and the aged population respectively), physical exercise (never exercised, less than 6 times a week, and more than 6 times a week), and educational level (above senior high school and senior high school and below).

### Statistical analysis

#### Description of the sample characteristics and EQ-5D reporting results

The participants were divided into self-report and proxy-report groups before PSM. Chi-squared tests were employed to compare group differences among socio-demographic factors, health behavior, and the dimensional results of EQ-5D-3 L. The relationships between the characteristics and the likelihood of proxy response were examined via Chi-squared test (categorical variables) and one-way ANOVA (continuous variables) to establish whether proxy was more likely to be used in some groups compared with others. A percent bar chart was adopted to summarize the original response results of 5 dimensions of EQ-5D-3 L for the overall population.

#### Implementation of PSM and balance checking

We speculated that the EQ-5D-3 L results of proxy-report and self-report respondents should be consistent when the two groups of respondents share the similar characteristics. Then, PSM was used to adjust their distribution of the main characteristics to the same level. After PSM, the difference of EQ-5D-3 L results between the two groups was regarded as proxy response bias. In PSM, proxy-report respondents were matched to self-report respondents with similar characteristics using a propensity score (PS), which was defined as the conditional probability of the individual being assigned to the proxy-report group. Multivariate logistic regression model was used to calculate PS. In the model, the dependent variable was the log of proxy and the independent variables were a set of conditioning variables, including socio-demographic factors, health behavior, and health status.

Nearest neighbor matching, which is one of the most widely used PSM methods, was chose in this study. The matching ratio was set to 1:2 for the following two reasons. First, before PSM, a total of 35,345 (80.1%) and 8789 (19.9%) respondents were restricted to self-report and proxy-report groups respectively. Then, we tried three matching ratios (1:2, 1:3 and 1:4) and the results showed that the number of unmatched cases in the treatment group was the least and the matching rate was the highest by 1:2 matching (see supplementary material for the matching results of 1:2, 1:3 and 1:4 matching). Second, Guo stated that 1:*n* matching was more efficient than 1:1 matching; however, when *n* was too large, it was impossible to allocate enough matched control group members for each treatment group member and the benefits of a large number of control group members were negligible [45]. The caliper width was set to 0.03 for the following two reasons. First, Austin PC recommended researchers to match on the logit of the propensity score using calipers of width equal to 0.2 of the standard deviation of the logit of the propensity score [46]. Second, the standard deviation of the logit of the propensity score in this study was 0.152369, and 0.2 times this value was approximately 0.03.

Standardized difference was used to check the balance of confounding factors after PSM. The balance of a confounder achieved once its standardized difference was lower than 10% after PSM [47]. PSM assumes that there remains no unobserved confounding. In this study, we use Harding’s approaches to test the underlying assumption of PSM that there remains no unobserved confounders [48] (see supplementary material for details about the method).

#### Analysis of proxy response effects

Proxy response effects were evaluated by using logistic regression model (conditional logistic model after PSM) and general linear model respectively to calculate the odds ratio (for EQ-5D-3 L dimensions) and mean differences (for EQ-5D-3 L index and EQ VAS), both with a 95% confidence interval (CI). In the logistic models, each of the 5 transformed EQ-5D-3 L dimensions was the dependent variable and the proxy indicator was the independent variable. In the general linear models, EQ-5D index or EQ VAS was used as the dependent variable while proxy indicator remained as the independent variable.

PSM was performed using the SPSS plug-in psmatching 3.02. Before and after PSM, all the statistical analysis was performed using SPSS 24.0. A difference of *P* <  0.05 was considered to be statistically significant.

## Results

Overall, 8789 (19.9%) intended respondents were reported by proxies. Table 1 presents the general characteristics of the sample and the difference between self-report and proxy-report groups before PSM. The group differences of the 13 covariates considered in Table 1 were all statistically significant (P <  0.05). In terms of the socio-demographic factors, the proxy-report group, compared with self-report group, showed a lower proportion of household heads, younger age, a higher proportion of unmarried and students, and a higher educational level. As for health behavior, the lifestyle of proxy-report group was much healthier with lower proportions of smoking and drinking and higher frequency of exercising. In addition, the health condition of proxy-report group was much better with lower proportions of participants who suffered from chronic diseases, were sick within two-week, and were hospitalized within 1 year.

**Table 1:** Basic characteristic of the sample at baseline

	Total	Proxy-report	Self-report	*P*
	n = (44134)	(*n* = 8789)	(*n* = 35,345)	
Household heads	18,884 (42.8)	1698 (19.3)	17,186 (48.6)	< 0.001
Age (y)				
15–44	18,282 (41.4)	5070 (57.7)	13,212 (37.4)	< 0.001
45–64	18,339 (41.6)	2216 (25.2)	16,123 (45.6)	
> 65	7513 (17.0)	1503 (17.1)	6010 (17.0)	
Male	22,610 (51.2)	4643 (52.8)	17,967 (50.8)	0.001
Marital status				
Unmarried	5497 (12.5)	2687 (30.6)	2810 (8.0)	< 0.001
Married	34,955 (79.2)	5265 (59.9)	29,690 (84.0)	
Widowed	3198 (7.2)	733 (8.3)	2465 (7.0)	
Divorced	436 (1.0)	83 (0.9)	353 (1.0)	
Others	48 (0.1)	21 (0.2)	27 (0.1)	
Senior high school and below	34,605 (78.4)	5942 (67.6)	28,663 (81.1)	< 0.001
In agriculture industry	36,825 (83.4)	7074 (80.5)	29,751 (84.2)	< 0.001
Employment status				
Employed	32,545 (73.7)	5246 (59.7)	27,299 (77.2)	< 0.001
Retiree	2756 (6.2)	478 (5.4)	2278 (6.4)	
Student	1999 (4.5)	1256 (14.3)	743 (2.1)	
Unemployed	6834 (15.5)	1809 (20.6)	5025 (14.2)	
Smoking status				
Smoker	13,742 (31.1)	2412 (27.4)	11,330 (32.1)	< 0.001
Ex-smoker	1316 (3.0)	202 (2.3)	1114 (3.2)	
Non-smoker	29,076 (65.9)	6175 (70.3)	22,901 (64.8)	
Alcohol consumption				
1–2 times a week	6563 (14.9)	1203 (13.7)	5360 (15.2)	< 0.001
≥ 3 times a week	1763 (4.0)	287 (3.3)	1476 (4.2)	
Non-drinker	35,808 (81.1)	7299 (83.0)	28,509 (80.7)	
Physical exercise				
≥ 6 times a week	4785 (10.8)	1004 (11.4)	3781 (10.7)	< 0.001
< 6 times a week	5636 (12.8)	1383 (15.7)	4253 (12.0)	
Never exercised	33,713 (76.4)	6402 (72.8)	27,311 (77.3)	
Chronic disease morbidity	10,152 (23.0)	1572 (17.9)	8580 (24.3)	< 0.001
Two-week morbidity rate	8621 (19.5)	1323 (15.1)	7298 (20.6)	< 0.001
Hospitalized	4191 (9.5)	682 (7.8)	3509 (9.9)	< 0.001

**Fig. 1:**
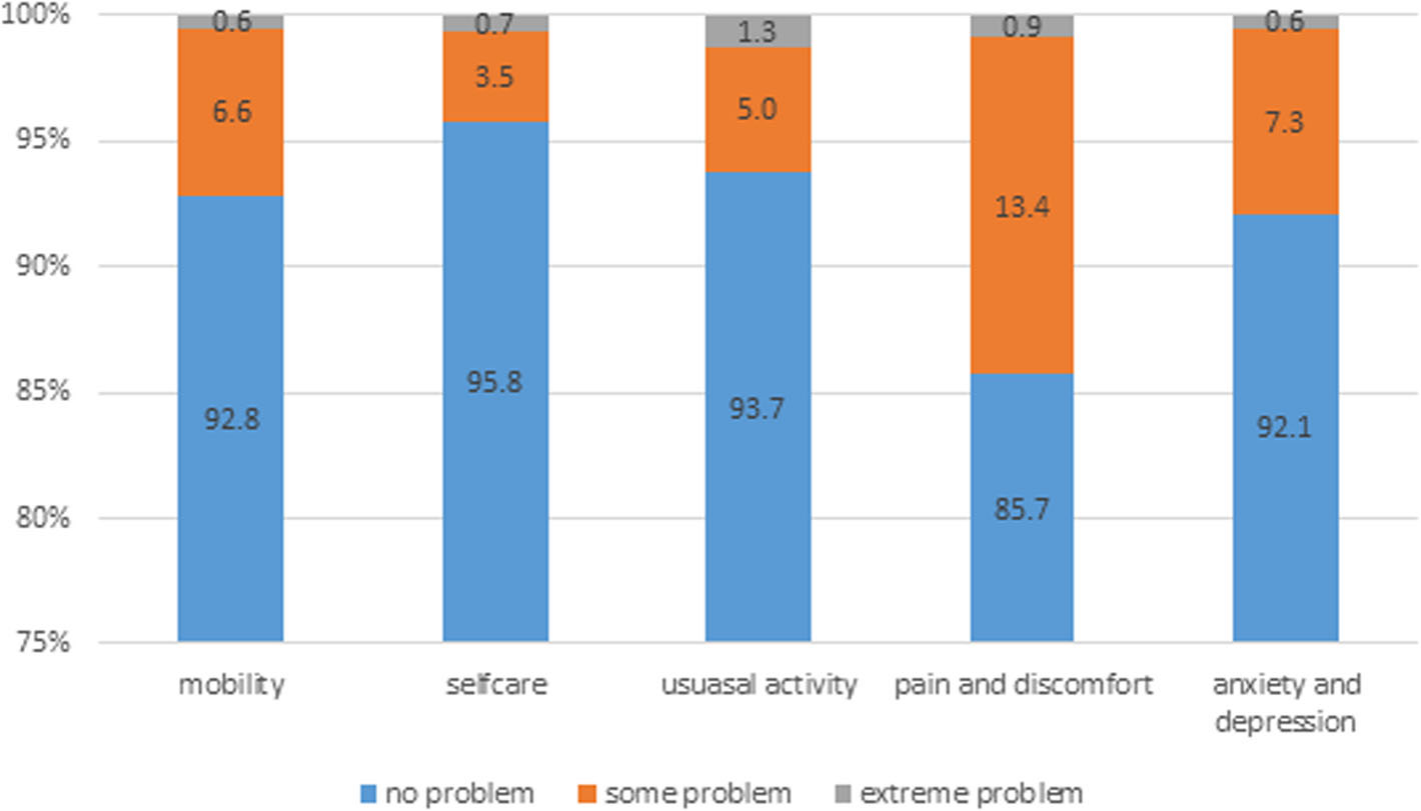
Results of EQ-5D-3 L in the overall population

In the overall population, the means of EQ-5D-3 L index and EQ VAS were 0.94 and 80.58 respectively. The original dimensional results are presented in Fig. 1. In general, the respondents reported health problems most in pain/discomfort dimension (14.3%) and least in self-care (4.2%). The proportion of reporting some problems in the pain/discomfort dimension was highest (13.4%), followed by anxiety/depression (7.3%), mobility (6.6%), usual activities (5.0%), and self-care (3.5%). The dimension reporting the most extreme problems was usual activities (1.3%), followed by self-care (0.9%).

**Fig. 2:**
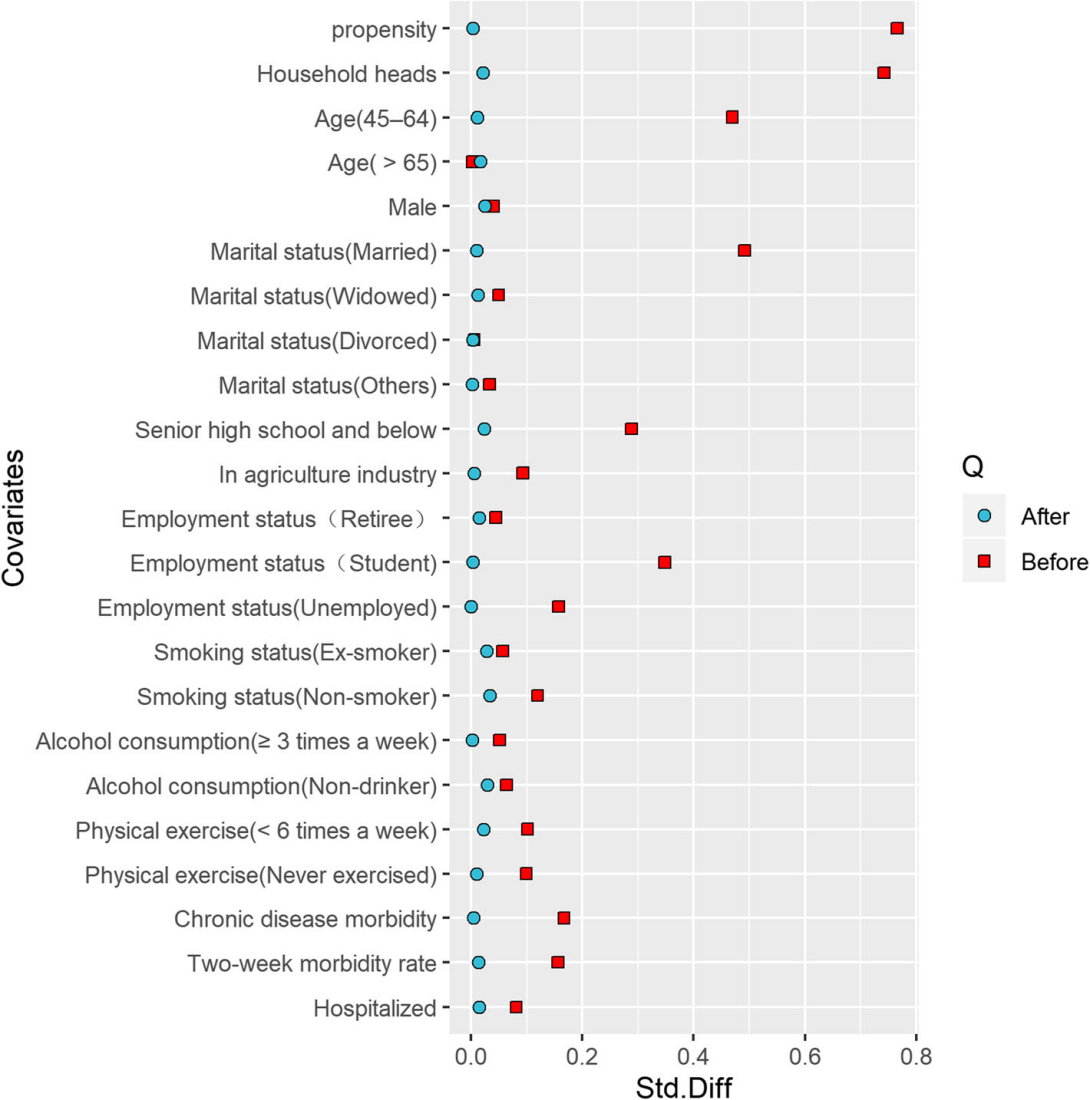
Absolute standardized differences before and after PSM

A total of 22,282 people were matched after PSM, of which 8190 were in the proxy-report group and 14,092 were in the self-report group. The between-group differences of all the covariates decreased and some of which, such as occupation (in agriculture industry), alcohol consumption, two-week morbidity, and hospitalization **(**Table 2**)**,were statistically non-significant with *P* values of Chi-squared tests larger than 0.05. Figure 2 shows that the standardized differences of all the 13 covariates are smaller than 10%, which indicates that PSM has really improved the between-group balance of the covariates.

**Table 2:** Comparison of baseline characteristics after PSM

	Proxy-report (*n* = 8190)	Self-report (*n* = 14,092)	*P*-value
Household heads	1698 (20.7)	3529 (25.0)	< 0.001
Age (y)			
15–44	4480 (54.7)	7107 (50.4)	< 0.001
45–64	2213 (27.0)	4267 (30.3)	
> 65	1497 (18.3)	2718 (19.3)	
Male	4284 (52.3)	7172 (50.9)	0.042
Marital status			
Unmarried	2104 (25.7)	2457 (17.4)	< 0.001
Married	5264 (64.3)	10,270 (72.9)	
Widowed	727 (8.9)	1208 (8.6)	
Divorced	83 (1.0)	142 (1.0)	
Others	12 (0.1)	15 (0.1)	
Senior high school and below	5857 (71.5)	10,394 (73.8)	< 0.001
In agriculture industry	6546 (79.9)	11,248 (79.8)	0.846
Employment status			
Employed	5208 (63.6)	9426 (66.9)	< 0.001
Retiree	478 (5.8)	970 (6.9)	
Student	729 (8.9)	736 (5.2)	
Unemployed	1775 (21.7)	2960 (21.0)	
Smoking status			
Smoker	2385 (29.1)	4265 (30.3)	0.002
Ex-smoker	199 (2.4)	432 (3.1)	
Non-smoker	5606 (68.4)	9395 (66.7)	
Alcohol consumption			
1–2 times a week	1174 (14.3)	2157 (15.3)	0.134
≥ 3 times a week	287 (3.5)	503 (3.6)	
Non-drinker	6729 (82.2)	11,432 (81.1)	
Physical exercise			
≥ 6 times a week	859 (10.5)	1518 (10.8)	0.009
< 6 times a week	1247 (15.2)	1936 (13.7)	
Never exercised	6084 (74.3)	10,638 (75.5)	
Chronic disease morbidity	1560 (19.0)	2931 (20.8)	0.002
Two-week morbidity	1304 (15.9)	2349 (16.7)	0.146
Hospitalized	674 (8.2)	1196 (8.5)	0.504

Tables 3 and 4 present the response results of EQ-5D for dimensions and scores respectively before and after PSM. Before PSM, proxy-report group was more likely to rate health problems in MO, SC, UA, and AD, except for PD (Table 3). The EQ-5D-3 L index of proxy-report group was 0.014 lower than that of self-report group, whilst the EQ VAS of the proxy-report group was 0.73 higher than that of the self-report group (Table 4). After PSM, the proportions of reporting any problems in the 5 dimensions were all significantly higher in the proxy-report group (Table 3). Compared with self-report group, all the odds ratios in the 5 dimensions were significantly larger than one, among which the largest dimension was SC, then followed by UA, MO, AD, and PD (Table 3). Simultaneously, proxy-report group got significantly lower means of EQ-5D-3 L index and EQ VAS (Table 4). These figures indicated that, after adjusting the measured confounding factors, consistent proxy response bias of EQ-5D-3 L among different constructs was identified, and the proxies were likely to underestimate the health status of the intended respondents, even though the magnitude of the proxy response bias varied greatly among different dimensions of EQ-5D-3 L and the difference of EQ-5D-3 L index and EQ VAS were relative low.

**Table 3:** Risk of proxy-report group to report any problems compared with self-report group before and after PSM

EQ-5D	Before PSM				After PSM			
	Proxy-report	Self-report	OR(95% CI)^a^	P value	Proxy-report	Self-report	OR(95% CI)^a^	*P*-value
Mobility	828 (9.4)	2351 (6.7)	1.46(1.34–1.59)	< 0.001	820 (10.0)	960 (6.8)	1.52(1.38–1.68)	< 0.001
Self-care	629 (7.2)	1224 (3.5)	2.15(1.95–2.37)	< 0.001	623 (7.6)	524 (3.7)	2.13(1.892–2.40)	< 0.001
Usual activity	841 (9.6)	1958 (5.5)	1.80(1.66–1.96)	< 0.001	833 (10.2)	837 (5.9)	1.79(1.622–1.98)	< 0.001
Pain/discomfort	1166 (13.3)	5144 (14.6)	0.90(0.84–0.96)	0.002	1157 (14.1)	1815 (12.9)	1.11(1.028–1.21)	0.008
Anxiety/depression	765 (8.7)	2719 (7.7)	1.14(1.05–1.24)	0.002	755 (9.2)	986 (7.0)	1.35(1.223–1.49)	< 0.001

^a^Self-reported group was the reference

**Table 4:** Means of EQ-5D index and VAS and the differences between proxy-report and self-report groups before and after PSM

Indicators	Before PSM				After PSM			
	Proxy-report	Self-report	Difference (95%CI)	*P*	Proxy-report	Self-report	Difference (95%CI)	*P*
EQ - 5D index^a^	0.929 (0.178)	0.943 (0.132)	−0.014(−0.018,-0.011)	< 0.001	0.946 (0.134)	0.924 (0.183)	−0.022(−0.026,-0.018)	< 0.001
EQ - VAS	81.16 (15.37)	80.43 (12.88)	0.73(0.417,1.043)	< 0.001	81.40 (13.20)	80.50 (15.50)	−0.83(−1.218,-0.450)	< 0.001

^a^The range of EQ-5D is 0–1. In order to express the small difference of EQ-5D index, three decimal numbers is retained

In the Harding’s approach, we supposed that there was unobserved binary confounder and specified the range of prevalence of the unobserved confounder among the self-reported group from 1 to 25%. The test results showed that the OR values of the five dimensions were very similar with those before being adjusted for unobserved confounding, indicating that the likelihood of reporting any health problems on each dimension was not sensitive to an unobserved confounder (see supplementary material for details about the method and results of sensitive analysis for unobserved confounding). Based on this evidence, we believe that it is reasonable to assume that there were no important confounders that remained uncontrolled for and thus the PSM was an appropriate method in this study.

## Discussion

Many studies have shown that the application of proxy response in the evaluation of objective and subjective health indicators would lead to selection bias [29]. This study try to evaluate the proxy response bias of Chinese EQ-5D-3 L in general population by using PSM and confirms that proxies are likely to report more health problems in EQ-5D-3 L and therefore leads to negative proxy response bias, which is consistent with most existing research findings. However, some of the results are inconsistent with those of previous studies.

First, the intended respondents of proxy-report group in this study were quite different from those in previous studies. In the existing studies, the intended respondents of proxy-report groups were mostly elderly people with diseases (such as disability, dementia, cognitive impairment, etc.) [16, 18, 21, 23–26, 28], while the proxy-report group in this study were younger and with lower prevalence of smoking, drinking, chronic disease, two-week morbidity and half-year hospitalization. The interviews were mainly conducted on weekdays, a time when most young and middle-aged migrant workers and students were not at home, especially in rural area, which may contribute to this age distribution difference.

Second, the magnitude of the proxy response bias among different dimensions of EQ-5D-3 L is inconsistent with most previous studies. After applying PSM, the proxy response bias sequence of the five dimensions was as follows: self-care, usual activity, mobility, anxiety/depression, and pain/discomfort. It suggested that the proxy response bias in physical dimensions (self-care, usual activity, mobility) was larger than those in psychological dimensions (anxiety/depression, pain/discomfort.), which was, however, contrary to the conclusions of existing studies [17, 20, 22]. These studies used kappa values to evaluate the agreement between self-report and proxy-report groups, and found that they showed more agreement on mobility, self-care, and usual activities than on pain/discomfort and anxiety/depression, which suggested that the proxy response bias of physical dimensions was smaller than that of psychological dimensions. Studies have also shown that the proxy response bias is most significant in psychological aspects and proxy responses are not suitable for the anxiety dimension [49, 50]. Similar to our findings, a study done in UK on residents aged > 85 years also reported highest proxy response bias in the self-care dimension [51].

The negative proxy response bias may be explained by the following reasons. First, if the proxies are in a poor the physical health status [50, 52, 53], it may be projected onto the HRQL evaluation of the intended respondents. Second, the proxy and the intended respondents may not in a fairly close relation. When spouses were proxies, as some studies suggested, the proxy response bias produced was less than that made by other types of proxies [49, 54, 55]. Third, the proxies may face great caregiver stress if he/she was a caregiver of the intended respondents [53, 56]. There are also some possible reasons for the characteristics of the magnitude of the proxy response bias among different dimensions. The self-care ability of intended respondents would directly affect the care burden of other family members. Therefore, other family members may overestimate the care services provided by them and report relatively poorer HRQL. However, the overestimation of health problems in the anxiety/depression and pain/discomfort dimensions by proxies is relatively low because the condition of intended respondents in these dimensions may not have a great effect on the care difficulty for the caregivers.

In this study, the EQ-5D-3 L index and EQ VAS were very high because the subjects of this study were selected from general population, who were more prone to report very low proportion of health problems. Therefore, the difference of the absolute values of EQ-5D-3 L index and EQ VAS between proxy-report and self-report groups was − 0.022 and − 0.834 respectively. It was very low and seemed to be meaningless. However, the proxy-reported group was 52, 113, 79, 11 and 35% more likely to report health problems on the five dimensions respectively, indicating very large relative difference. In addition, the proxy response rate of NHSS is as high as 19.9%. Thus, it is necessary to adjust proxy response bias of EQ-5D-3 L in studies based on NHSS data. This study is the first time to analyze the proxy response effect of Chinese version of EQ-5D-3 L based on NHSS data, which is of great significance for the evaluation of proxy response bias in Chinese EQ-5D-3 L and provide valuable knowledge for the application of Chinese EQ-5D-3 L in HTA in the future.

### Study limitations

This study had several limitations. First, the study use proxies mostly for the reason that the intended respondents are not at home, rather than unable to report, which suggests that the proxies are in most cases family members, not necessarily caregivers. Therefore, it is difficult to verify the proxies’ familiarity with the health status of the intended respondents, and the rationality and validity of the using of proxies need to be further studied. Second, the information about the role of the proxy was not collected, therefore the effect of the proxy type could not be analyzed. Third, the EQ-5D-3 L results of the matched pairs are not from the exactly same respondent. Future studies with paired design in which both the proxies and the intended respondents report the proxy version of EQ-5D-3 L are needed to determine the “real” proxy bias in general population.

## Conclusions

Significant negative proxy response bias was found in Chinese version of EQ-5D-3 L in general population, and the magnitude of the bias was larger in physical dimensions than psychological dimensions by using PSM to control confounders. It provided valuable knowledge for the application of Chinese EQ-5D-3 L in HTA in the future.

## Supplementary information

**Additional file 1.** Method and results of sensitive analysis for unobserved confounding.

## Data Availability

Not applicable.

## Abbreviations

AC: Usual activities
AD: Anxiety/depression
BRFSS: Behavioral risk factor surveillance system
CI: Confidence interval
HRQL: Health-related quality of life
MO: Mobility
NHIS: National Health Interview Survey
NHSS: National Health Service Survey
OR: Odds ratio
PD: Pain/discomfort
PSM: Propensity score matching
RCT: Randomized controlled trials
SC: Self-care
VAS: Visual analogue scale
