# Supplementary material for: Assessing the proxy response bias of EQ–5D-3 L in general population: a study based on a large-scale representative household health survey using propensity score matching
Source: Health Qual Life Outcomes. 2020 Mar 18;18:75. doi: 10.1186/s12955-020-01325-z (PMC7079393; doi:10.1186/s12955-020-01325-z)
Supplement: Supplementary file 1 — Additional file 1. Method and results of sensitive analysis for unobserved confounding. [file 12955_2020_1325_MOESM1_ESM.docx]

**1. The results of 1:2, 1:3 and 1:4 matching**

Results of matching with different ratios

|  | 1：2 | |  | 1：3 | |  | 1：4 | |
| --- | --- | --- | --- | --- | --- | --- | --- | --- |
|  | Control | Treated |  | Control | Treated |  | Control | Treated |
| All | 35345 | 8789 |  | 35345 | 8789 |  | 35345 | 8789 |
| Matched | 14092 | 8190 |  | 18602 | 8182 |  | 21811 | 8188 |
| Unmatched | 21253 | 599 |  | 16743 | 607 |  | 13534 | 601 |

**2. Harding’s approach**

When performing sensitivity analyses, researchers generally specify a range of parameter values either suggested by the literature or based on the relationships between observed confounders and the exposure and outcome to examine the sensitivity of study inferences under different specifications. This approach is particularly useful when it is not easy to obtain some parameters that require outside knowledge. Therefore, we specify a single set of parameters to be used in the motivating example.

**The following notation will be used:**

*x* = binary treatment status/exposure

*y* = binary outcome

*u* = unobserved binary confounder

*c* = observed confounders

*p*(*x*) = prevalence of the exposure

*p*(*u*) = prevalence of the unobserved confounder

*p*(*u*|*x*=1) = prevalence of the unobserved confounder among the exposed group

*p*(*u*|*x*=0) = prevalence of the unobserved confounder among the unexposed group

OR*yu* = odds ratio of the relationship between the outcome and unobserved confounder

OR*xu* = odds ratio of the relationship between the exposure and unobserved confounder

OR*yx• c* = observed odds ratio of the relationship between the outcome and the exposure from the data, adjusted for c (but not for u)

OR*yx•cu* = true (bias-free/bias-adjusted) odds ratio of the relationship between the outcome and the exposure, adjusted for both c and u

Hardly can we identify the relationship between the hypothetical unobserved confounder (*_u_*) and the exposure (OR*_xu_*) and the outcome (OR*_yu_*) (net of all the covariates matched on) from the existing literature, so we examined the relationships of the observed confounders (c) in this study with the exposure (OR_xc_) and the outcome (OR*_yc_*). The result shows that the ORs between the observed confounders and the exposure ranged from 0.678 to 1.288, with five dimensions ofORs between the observed confounder and the outcome (y) ranging respectively 0.440-12.850, 0.149-11.968, 0.107-10.591, 0.449-7.082 and 0.316-2.589. To err on the conservative side, we fixed the values of OR*_xu_* and OR*_yu_* at these two highest values of the observed OR’s (i.e., OR*_xu_*=1.288; OR*_yu_*_-MO_=12.850; OR*_yu_*_-SC_=11.968; OR*_yu_*_-AC_=10.591; OR*_yu_*_-PD_=7.089; OR*_yu_*_-AD_=2.589). Some approaches require the specification of *p*(*u*|*x*=1)and *p*(*u*|*x*=0) instead of OR*_xu_*. The prevalence of a hypothetical unobserved confounder in the general population is not available; hence, we specified a range of *p*(*u*|*x*=0) from 1% to 25% with the fixed OR*_xu_*=1.288 to obtain varying *p*(*u*|*x*=1).

***Settings and inputs***

- Allow any study design
- Suitable for binary outcomes only
- OR*_xu_* and OR*_yu_*

| Dimensions | OR*_yu_* (Δ) | OR*_xu_* (Γ) |
| --- | --- | --- |
| MO | 12.850 | 1.288 |
| SC | 11.968 | 1.288 |
| AC | 10.591 | 1.288 |
| PD | 7.089 | 1.288 |
| AD | 2.589 | 1.288 |

- 2x2 table of observed *x* and *y*

|  |  | MO | |
| --- | --- | --- | --- |
|  |  | Y=0 | Y=1 |
| MO | *x*=0 | *a*+*e*=13132 | *b*+*f*=960 |
|  | *x* =1 | *c*+*g*=7370 | *d*+*h*=820 |
| SC | *x* =0 | *a*+*e*=13568 | *b*+*f*=524 |
|  | *x* =1 | *c*+*g* =7567 | *d*+*h*=623 |
| AC | *x* =0 | *a*+*e*=13255 | *b*+*f*=837 |
|  | *x* =1 | *c*+*g*=7357 | *d*+*h*=833 |
| PD | *x* =0 | *a*+*e*=12277 | *b*+*f* =1815 |
|  | *x* =1 | *c*+*g*=7033 | *d*+*h*=1157 |
| AD | *x* =0 | *a*+*e*=13106 | *b*+*f*=986 |
|  | *x* =1 | *c*+*g*=7435 | *d*+*h*=755 |

OR*_yu_*=*af*/*be*=*ch*/*dg*

OR*_xu_*=*ag*/*ce*=*bh*/*fd* Equation 1

*p(u)* *(*a*+*b*+*c*+*d*)=(1- *p(u)*) *(*e*+*f*+*g*+*h*)

*p(u|x=0)* = 0.01: 0.0114*(*a*+*b*+*c*+*d*)=0.9886*(*e*+*f*+*g*+*h*)^a^

*p(u|x=0)* = 0.25: 0.2855*(*a*+*b*+*c*+*d*)=0.7145*(*e*+*f*+*g*+*h*)^a^

^a^ The ratio of *a+b+c+d* and *e+f+g+h* was pre-specified by using *p(x)* = 0.50, *p(u|x=0)* = 0.01, *p(u|x=1)* = 0.0128 (obtained from *ORxu*=1.288 and *p(u|x=0)* = 0.01 using Equation 2) to obtain *p(u)* = 0.0114, and by using *p(x)* = 0.50, *p(u|x=0)* = 0.25, *p(u|x=1)* = 0.0128 (obtained from *ORxu*=1.288 and *p(u|x=0)* = 0.25 using Equation 2) to obtain *p(u)* = 0.2855.

$p\left( u | x=1 \right)=\frac{{OR}_{xu}*p(u|x=0)}{1-p\left( u | x=0 \right)+ORxu*p(u|x=0)}$ Equation 2

***Method: hand computation***

**i. Use an online system solver to find the cell count *a-h* using Equation 1**

<https://www.solvemymath.com/online_math_calculator/algebra_combinatorics/system_of_equations/index.php>

**ii. Enter *a-h* into the 2 by 2 table**

| p(u\|x=0) | Dimension |  |  | *y*=0 | *y*=1 |
| --- | --- | --- | --- | --- | --- |
| 0.01 | MO | *u*=0 | *x*=0 | a=13060.6 | b=897.0 |
|  |  |  | *x* =1 | c=7318.5 | d=752.0 |
|  |  | *u*=1 | *x*=0 | e=71.4 | f=63.0 |
|  |  |  | *x* =1 | g=51.5 | h=68.0 |
|  | SC | *u*=0 | *x*=0 | a=13477.1 | b=484.8 |
|  |  |  | *x* =1 | c=7501.8 | d=564.3 |
|  |  | *u*=1 | *x*=0 | e=90.9 | f=39.2 |
|  |  |  | *x* =1 | g=65.2 | h=58.7 |
|  | AC | *u*=0 | *x*=0 | a=13174.1 | b=785.9 |
|  |  |  | *x* =1 | c=7299.3 | d=768.6 |
|  |  | *u*=1 | *x*=0 | e=80.9 | f=51.1 |
|  |  |  | *x* =1 | g=57.7 | h=64.4 |
|  | PD | *u*=0 | *x*=0 | a=12205.9 | b=1743.0 |
|  |  |  | *x* =1 | c=6980.6 | d=1098.5 |
|  |  | *u*=1 | *x*=0 | e=71.1 | f=72.0 |
|  |  |  | *x* =1 | g=52.4 | h=58.5 |
|  | AD | *u*=0 | *x*=0 | a=12985.5 | b=962.9 |
|  |  |  | *x* =1 | c=7347.2 | d=732.3 |
|  |  | *u*=1 | *x*=0 | e=120.5 | f=23.1 |
|  |  |  | *x* =1 | g=87.8 | h=22.7 |
| 0.25 | MO | *u*=0 | *x*=0 | a=10197.1 | b=204.3 |
|  |  |  | *x* =1 | c=5376.8 | d=142.3 |
|  |  | *u*=1 | *x*=0 | e=2934.9 | f=755.7 |
|  |  |  | *x* =1 | g=1993.2 | h=677.7 |
|  | SC | *u*=0 | *x*=0 | a=10319.9 | b=109.9 |
|  |  |  | *x* =1 | c=5384.3 | d=106.5 |
|  |  | *u*=1 | *x*=0 | e=3248.2 | f=414.1 |
|  |  |  | *x* =1 | g=2182.7 | h=516.5 |
|  | AC | *u*=0 | *x*=0 | a=10226.9 | b=202.4 |
|  |  |  | *x* =1 | c=5325.9 | d=165.3 |
|  |  | *u*=1 | *x*=0 | e=3028.1 | f=634.6 |
|  |  |  | *x* =1 | g=2031.1 | h=667.7 |
|  | PD | *u*=0 | *x*=0 | a=9706.7 | b=630.8 |
|  |  |  | *x* =1 | c=5244.4 | d=338.5 |
|  |  | *u*=1 | *x*=0 | e=2570.3 | f=1184.2 |
|  |  |  | *x* =1 | g=1788.6 | h=818.5 |
|  | AD | *u*=0 | *x*=0 | a=9830.9 | b=529.4 |
|  |  |  | *x* =1 | c=5202.6 | d=357.7 |
|  |  | *u*=1 | *x*=0 | e=3275.1 | f=456.6 |
|  |  |  | *x* =1 | g=2232.4 | h=397.3 |

**iii. These 8 cell counts are employed as frequency weights to re-create a dataset that contains information on the unobserved confounder.**

| p(u\|x=0) |  | x | y | u | # of combinations (weights) | | | | |
| --- | --- | --- | --- | --- | --- | --- | --- | --- | --- |
|  |  |  |  |  | MO | SC | AC | PD | AD |
| 0.01 | a | 0 | 0 | 0 | 13060.6 | 13477.1 | 13174.1 | 12205.9 | 12985.5 |
|  | b | 0 | 1 | 0 | 897.0 | 484.8 | 785.9 | 1743.0 | 962.9 |
|  | c | 1 | 0 | 0 | 7318.5 | 7501.8 | 7299.3 | 6980.6 | 7347.2 |
|  | d | 1 | 1 | 0 | 752.0 | 564.3 | 768.6 | 1098.5 | 732.3 |
|  | e | 0 | 0 | 1 | 71.4 | 90.9 | 80.9 | 71.1 | 120.5 |
|  | f | 0 | 1 | 1 | 63.0 | 39.2 | 51.1 | 72.0 | 23.1 |
|  | g | 1 | 0 | 1 | 51.5 | 65.2 | 57.7 | 52.4 | 87.8 |
|  | h | 1 | 1 | 1 | 68.0 | 58.7 | 64.4 | 58.5 | 22.7 |
| 0.25 | a | 0 | 0 | 0 | 10197.1 | 10319.9 | 10226.9 | 9706.7 | 9830.9 |
|  | b | 0 | 1 | 0 | 204.3 | 109.9 | 202.4 | 630.8 | 529.4 |
|  | c | 1 | 0 | 0 | 5376.8 | 5384.3 | 5325.9 | 5244.4 | 5202.6 |
|  | d | 1 | 1 | 0 | 142.3 | 106.5 | 165.3 | 338.5 | 357.7 |
|  | e | 0 | 0 | 1 | 2934.9 | 3248.2 | 3028.1 | 2570.3 | 3275.1 |
|  | f | 0 | 1 | 1 | 755.7 | 414.1 | 634.6 | 1184.2 | 456.6 |
|  | g | 1 | 0 | 1 | 1993.2 | 2182.7 | 2031.1 | 1788.6 | 2232.4 |
|  | h | 1 | 1 | 1 | 677.7 | 516.5 | 667.7 | 818.5 | 397.3 |

**iv. By using this re-created data, a weighted logistic regression can be performed to obtain OR*yx•cu*, including its confidence interval.**

| **p(u\|x=0)** | **Dimension** | **Variable** | **B** | **Sig.** | **Exp(B)** | **95.0% C.I.for Exp(B)** | |
| --- | --- | --- | --- | --- | --- | --- | --- |
|  |  |  |  |  |  | **Lower** | **Upper** |
| 0.01 | MO | Intercept | 1.200 | <0.001 |  |  |  |
|  |  | x | 0.201 | <0.001 | **1.496** | **1.355** | **1.652** |
|  |  | u | 1.277 | <0.001 | 12.847 | 9.982 | 16.536 |
|  | SC | Intercept | 1.715 | <0.001 |  |  |  |
|  |  | x | 0.369 | <0.001 | **2.091** | **1.852** | **2.360** |
|  |  | u | 1.241 | <0.001 | 11.968 | 9.189 | 15.587 |
|  | AC | Intercept | 1.355 | <0.001 |  |  |  |
|  |  | x | 0.284 | <0.001 | **1.765** | **1.595** | **1.953** |
|  |  | u | 1.180 | <0.001 | 10.591 | 8.209 | 13.664 |
|  | PD | Intercept | 0.918 | <0.001 |  |  |  |
|  |  | x | 0.049 | 0.017 | **1.102** | **1.017** | **1.194** |
|  |  | u | 0.979 | <0.001 | 7.089 | 5.525 | 9.096 |
|  | AD | Intercept | 1.978 | <0.001 |  |  |  |
|  |  | x | 0.148 | <0.001 | **1.344** | **1.217** | **1.484** |
|  |  | u | 0.476 | <0.001 | 2.591 | 1.874 | 3.584 |
| 0.25 | MO | Intercept | 2.494 | <0.001 |  |  |  |
|  |  | x | 0.139 | <0.001 | **1.321** | **1.190** | **1.465** |
|  |  | u | 1.277 | <0.001 | 12.850 | 11.376 | 14.514 |
|  | SC | Intercept | 2.992 | <0.001 |  |  |  |
|  |  | x | 0.309 | <0.001 | **1.856** | **1.639** | **2.102** |
|  |  | u | 1.241 | <0.001 | 11.967 | 10.285 | 13.925 |
|  | AC | Intercept | 2.518 | <0.001 |  |  |  |
|  |  | x | 0.225 | <0.001 | **1.569** | **1.412** | **1.743** |
|  |  | u | 1.180 | <0.001 | 10.591 | 9.391 | 11.945 |
|  | PD | Intercept | 1.757 | <0.001 |  |  |  |
|  |  | x | -0.003 | 0.875 | *0.993* | *0.913* | *1.081* |
|  |  | u | 0.979 | <0.001 | 7.088 | 6.518 | 7.709 |
|  | AD | Intercept | 2.324 | <0.001 |  |  |  |
|  |  | x | 0.122 | <0.001 | **1.277** | **1.155** | **1.411** |
|  |  | u | 0.476 | <0.001 | 2.589 | 2.345 | 2.859 |

**v. Interpretation**

Almost all the values of ORyx•cu (except for PD dimension when *p*(*u*|*x*=0) is pre-specified to be 0.25) adjusted, considering the unobserved confounders are still statistically significantly larger than 1 and very similar to those without adjustment, suggesting that the results is not sensitive to an unobserved confounder. Based on this, we believe that it is reasonable to assume that no important confounders remained uncontrolled, thus PSM is an appropriate method in this study.
